# Loop Mediated Isothermal Amplification (LAMP) Accurately Detects Malaria DNA from Filter Paper Blood Samples of Low Density Parasitaemias

**DOI:** 10.1371/journal.pone.0103905

**Published:** 2014-08-08

**Authors:** Berit Aydin-Schmidt, Weiping Xu, Iveth J. González, Spencer D. Polley, David Bell, Delér Shakely, Mwinyi I. Msellem, Anders Björkman, Andreas Mårtensson

**Affiliations:** 1 Malaria Research, Department of Microbiology, Tumor and Cellbiology, Karolinska Institutet, Stockholm, Sweden; 2 Department of Clinical Parasitology, Foundation for Innovative New Diagnostics (FIND), Geneva, Switzerland; 3 Hospital for Tropical Diseases, University College London Hospitals, London, United Kingdom; 4 NIHR Biomedical Research Centre at University College London Hospitals NHS Foundation Trust and University College, London, United Kingdom; 5 Global Good Fund, Bellevue, Washington, United States of America; 6 Global Health, Department of Public Health Sciences, Karolinska Institutet, Stockholm, Sweden; 7 Zanzibar Malaria Elimination Program, Ministry of Health, Zanzibar, Tanzania; 8 Unit of infectious diseases, Karolinska University Hospital, Stockholm, Sweden; 9 Center for Clinical Research, Sörmland County Council, Sörmland, Sweden; Université Pierre et Marie Curie, France

## Abstract

**Background:**

Loop mediated isothermal amplification (LAMP) provides an opportunity for improved, field-friendly detection of malaria infections in endemic areas. However data on the diagnostic accuracy of LAMP for active case detection, particularly low-density parasitaemias, are lacking. We therefore evaluated the performance of a new LAMP kit compared with PCR using DNA from filter paper blood spots.

**Methods and Findings:**

Samples from 865 fever patients and 465 asymptomatic individuals collected in Zanzibar were analysed for Pan (all species) and Pf (*P. falciparum*) DNA with the Loopamp MALARIA Pan/Pf kit. Samples were amplified at 65°C for 40 minutes in a real-time turbidimeter and results were compared with nested PCR. Samples with discordant results between LAMP and nested PCR were analysed with real-time PCR. The real-time PCR corrected nested PCR result was defined as gold standard. Among the 117 (13.5%) PCR detected *P. falciparum* infections from fever patients (mean parasite density 7491/µL, range 6–782,400) 115, 115 and 111 were positive by Pan-LAMP, Pf-LAMP and nested PCR, respectively. The sensitivities were 98.3% (95%CI 94–99.8) for both Pan and Pf-LAMP. Among the 54 (11.6%) PCR positive samples from asymptomatic individuals (mean parasite density 10/µL, range 0–4972) Pf-LAMP had a sensitivity of 92.7% (95%CI 80.1–98.5) for detection of the 41 *P. falciparum* infections. Pan-LAMP had sensitivities of 97% (95%CI 84.2–99.9) and 76.9% (95%CI 46.2–95) for detection of *P. falciparum* and *P. malariae*, respectively. The specificities for both Pan and Pf-LAMP were 100% (95%CI 99.1–100) in both study groups.

**Conclusion:**

Both components of the Loopamp MALARIA Pan/Pf detection kit revealed high diagnostic accuracy for parasite detection among fever patients and importantly also among asymptomatic individuals of low parasite densities from minute blood volumes preserved on filter paper. These data support LAMPs potential role for improved detection of low-density malaria infections in pre-elimination settings.

## Background

In areas of sub-Saharan Africa where malaria elimination is targeted, new tools and strategies are needed to achieve this ambitious goal. A critical component to pursue malaria elimination is to identify and treat all malaria parasite carriers, both symptomatic patients and asymptomatic individuals through passive and active case detection, respectively [1]. Importantly, during the transition from malaria control to pre-elimination and eventually elimination, the relative importance of low-density parasitaemias as a reservoir of on-going transmission is expected to gradually increase [2], [3], [4]. Conventional point-of-care malaria diagnostic tools, i.e. microscopical investigation of stained blood smears and rapid diagnostic tests (RDTs), are not sensitive enough for reliable detection of low-density parasitaemias, considering their limit of detection of approximately 100 parasites/µL of blood in field use [5], [6], [7]. Conversely, highly sensitive diagnostic tools like PCR are hampered by their complexity, need for specialized laboratory infrastructure and know-how, relatively long time-to-result and thus lack of field friendliness [8].

Newly described molecular based diagnostic methods, such as loop-mediated isothermal amplification of DNA (LAMP), provide an opportunity to raise the target of both efficient passive and active malaria case detection to include also low-density parasitaemias in field settings of endemic areas [2], . The new Loopamp MALARIA Pan/Pf detection kit ((LMC 562, Eiken Chemical Co., Ltd. Tokyo, Japan) consists of two separate reagents tubes containing vacuum dried reaction mixtures with primers targeting the mitochondrial DNA sequences of all human *Plasmodium* species (Pan-malaria) and *Plasmodium falciparum* (Pf), respectively. The kit is designed to address the need for a molecular test that achieves higher sensitivity and specificity than microscopy and RDT and at the same time it is more field-friendly than PCR since it employs temperature stable vacuum dried reagents requiring no cold transport chain, is easily reconstituted on site and neither requires a thermocycler for amplification nor a gel imaging system for result reading [5], [10]. The *Bacillus stearothermophilus* (*Bst*) polymerase used in the LAMP method is also considered more robust than *Taq* polymerase with regards to inhibition of the reaction, making it suitable for simple and rapid DNA extraction methods [11]. A feasibility study has previously demonstrated that the Loopamp MALARIA Pan/Pf kit can amplify *Plasmodium* DNA in less than 40 minutes from samples containing as low as 2 parasites per microliter (p/µL) of blood, with a sensitivity and specificity of 93.3% and 100%, respectively, using nested PCR as reference standard [12]. A recent field study conducted in a high transmission area of Uganda using venous blood samples from fever patients has also shown that technicians without previous molecular training could reliably perform LAMP in a simple laboratory space without specialized equipment after a short training period [5]. Under these conditions LAMP had sensitivity and specificity equivalent to nested PCR performed upon paired samples in a reference level laboratory, but with a significantly faster time-to-result.

Both these studies employed liquid blood from venipuncture [12]. However, convincing evidence that the LAMP method can reliably detect parasite DNA extracted from finger prick blood samples spotted and dried onto filter paper from asymptomatic carriers of low parasite densities (necessary for an effective field screening programme) is lacking. Moreover, the performance of LAMP for overall improved malaria case detection in low-endemic/malaria pre-elimination settings remains to be shown. The aim of this study was therefore to evaluate the accuracy of the Loopamp MALARIA Pan/Pf kit as a comparator to PCR for detection of malaria parasite carriers in Zanzibar, a malaria pre-elimination setting of sub-Saharan Africa. Samples were derived from archived dried blood spots collected both from fever patients attending primary health care facilities and asymptomatic individuals participating in a cross-sectional survey.

## Methods

### Study design, blood samples and their origin

This comparative study on the diagnostic accuracy of the Loopamp MALARIA Pan/Pf detection kit for detection of pan-*Plasmodium* and *P. falciparum* DNA, respectively, versus PCR as reference standard, included a total of 1330 archived blood samples collected on filter papers, of which 865 originated from fever patients and 465 from asymptomatic individuals in Zanzibar.

The study was conducted according to the STARD (Standards for reporting of Diagnostic Accuracy Studies) guidelines [13].

#### Fever patients

The 865 blood samples were collected from fever patients presenting at primary health care facilities in two districts, i.e. North A (Unguja Island) and Micheweni (Pemba Island), in Zanzibar, 2010. All patients were enrolled in a previously published malaria RDT study [14]. Briefly, 3890 fever patients (defined as either documented axillary temperature ≥37.5°C at enrolment or history of fever during the preceding 24 hours) aged ≥2 months were tested for *P. falciparum* malaria with a histidine rich protein 2 (HRP2) detecting RDT (Paracheck-Pf,Orchid Biomedical Systems, India). Overall, 121 (3.1%) patients were RDT positive. Blood smears for microscopy and a blood spot on filter paper (Whatman™3MM, GE Health care, UK) for molecular analysis were collected from capillary finger pricks, from all RDT positive patients and 744 (∼20%) randomly selected RDT negative patients. All RDT positive samples were previously analysed with three standard *P. falciparum* specific nested PCR methods [14], whereas the RDT negative samples were screened in duplicate for human plasmodial infection with an 18 s real-time PCR [15]. In total 122 of the 865 (121 RDT positive+744 RDT negative) patients were PCR positive for *P. falciparum*. No other species was found. Four of the 121 (3.3%) RDT positive patients were negative by PCR, whereas five of the 744 (0.7%) RDT negative patients were positive by PCR [14]. All samples were analysed with Cyt b nested PCR in association with the LAMP assays.

#### Asymptomatic individuals

The 465 blood samples were selected from asymptomatic individuals participating in a community based cross sectional survey conducted in North A and Micheweni districts in Zanzibar, 2011. A total of 2977 individuals were screened with a *Pf*HRP2/pan-*Plasmodium* lactate dehydrogenase (pLDH) based RDT (SD-BioLine Malaria Ag P.f/Pan,Standard Diagnostic, Inc, Republic of Korea). Blood was also collected on filter paper (Whatman 3MM) and subsequently analysed with a nested [16] and a real-time PCR, both targeting the Cytochrome b (Cyt b) gene. The RDT positivity rate was 13/2977 (0.4%), of which 6 (46%) samples were confirmed positive by PCR, all *P. falciparum* mono- infections. The corresponding overall PCR positivity rate was 65/2977 (2.2%), 61 (93.8%) were detected by nested PCR and additionally four by real-time PCR. All 65 PCR positive samples and 400 randomly selected PCR negative samples were included in the present analysis. Prior to the present study, DNA was re-extracted with the Chelex method from all 65 original PCR positive samples due to insufficient amount of remaining DNA.

### Molecular analyses

#### DNA extraction from dried blood spots on filter paper

*Fever patients*: DNA was extracted from three Ø 3 mm filter paper punches, equivalent to approximately 10–15 µL blood, using a modified version of the column-based ABI 6100 Nucleic Acid Prep Station protocol (Applied Biosystems, Fresno, CA) [17]. Each DNA sample was eluted in 200 µL of buffer and stored in −20°C until use.

*Asymptomatic individuals*: DNA was extracted from one Ø 3 mm filter paper punch, equivalent to approximately 3–5 µL blood. Each filter paper was suspended in 0.5% saponin PBS buffer (Sigma, St Louis, MO), washed in 1× PBS buffer (Gibco, Paisley, UK), boiled in 100 µL of 10% Chelex (Bio-Rad, Hercules, CA) H_2_O solution at 95°C for 10 min and centrifuged [18]. The supernatant containing DNA was obtained and transferred to a new plate, and stored at −20°C before further molecular analysis.

#### PCR methods

*Nested PCR:* Cyt b nested PCR as described by Steenkeste et al, 2009 [16] was performed on all samples prior to the LAMP assay. Briefly, primer pairs of 5′-TAATGCCTAGACGTA TTCCTGATTATCCAG-3′/5′-TGTTTGCTTGGGAGC TGTAATCATAATGTG-3′ and 5′-GAGAATTATGGAGT GGATGGTG-3′/5′-TGGTAATTGA CATCCAATCC-3′ were used in the first and nested PCR, respectively. PCR master mix was prepared [16] using 5 µL of extracted DNA and PCRs was run on the ABI Thermal Cycler 2700 using the same cycling conditions for the first and nested PCR runs except for the extension step. The conditions were 95°C for 3 min; 40 cycles of 95°C for 15 sec, 60°C for 1 min and 72°C for 1.5 min (first PCR) or 1 min (nested PCR); and final extension at 72°C for 5 min. The nested PCR products were visualized under UV light after gel electrophoresis, and positive PCR products were subjected to a Restriction Fragment Length Polymorphism (RFLP) assay for species identification [19]. The detection limit for Cyt b nested PCR was estimated to approximately 2 p/µL as assessed by *P. falciparum* 3D7 laboratory culture dilution series seeded on filter papers.

*Real-time PCR:* A recently developed Cyt b real-time PCR was performed on the Pan and/or Pf-LAMP/nested PCR discordant samples, using the primer pairs of 5′-TGGTAGCACAAATCCTTTAGGG-3′ and 5′-TGGTAATTGACATCCAATCC-3′ targeting the Cyt b gene of the five human *Plasmodia* species (unpublished data). The real-time PCR master mix was prepared containing 5 µL of extracted DNA, 1× iTaq Universal SYBR Green Supermix (Bio-Rad Laboratories, Hercules, CA), and 0.25 µM of each primer in 20 µL volume, and run on the ABI Prism 7000 system. The real-time PCR conditions were 95°C for 4 min; 40 cycles of 95°C for 15 sec, 60°C for 1.5 min with fluorescence detection; 72°C for 5 min; and melting curve acquisition. The real-time PCR results were analysed by both the melting curve and gel electrophoresis. Positive real-time PCR products were digested by FspBI enzyme (Thermo Fisher, Waltham, MA) in RFLP assay for species identification.The RFLP reaction was carried out in a total reaction volume of 20 µL including 5 µL of real-time PCR products and 5 units of FspBI enzyme in 1× reaction buffer in accordance with manufacture instructions. After overnight digestion in 37°C, the RFLP products were run on 2% agarose gel followed by visualization in Gel-doc system (Bio-Rad). The detection limit for Cyt b real-time PCR was estimated to approximately 1 p/µL as assessed by *P. falciparum* 3D7 laboratory culture dilution series seeded on filter papers.

#### Quantitative PCR determined parasite densities

All nested PCR positive samples from asymptomatic individuals were subjected to parasite density determination by *P. falciparum* 3D7 dilutions and quantitative PCR (qPCR) targeting the *Plasmodium* 18S rRNA gene [20]. Firstly, filter papers (Whatman 3MM) were prepared by diluting laboratory cultured *P. falciparum* 3D7 to densities of 20000, 2000, 200, 20 and 2 p/µL, spotting 30 µL from each density in the dilution series on to a separate filter papers and air dried. Secondly, filter papers were subjected to Chelex extraction and qPCR quantification targeting 18S rRNA gene using plasmid standards. Thirdly, a standard curve was developed by plotting known density to quantified 18S copy numbers. Fourthly, asymptomatic DNA samples were quantified by the 18S qPCR using plasmid standard [20], and the parasite densities were calculated by the acquired 18S copy numbers and the standard curve.

### LAMP procedures

All DNA samples were analysed with the Loopamp MALARIA Pan/Pf detection kit according to standard operating procedures provided by FIND and the manufacturer's instructions (available at: http://www.finddiagnostics.org/export/sites/default/programs/malaria-afs/docs/SOPs_LAMP_Malaria_AUG12.pdf) [21]. Samples were analysed individually for Pan and Pf using separate reaction-tubes containing specific primers. In brief, 30 µL of DNA samples (diluted 1∶6) were added to each Pan and Pf reaction tube. Each set of six samples were analysed along with a negative and a positive control included in the kit. After mixing the DNA solution with the dried reagents, the LAMP reaction tubes were incubated at 65°C for 40 minutes followed by a 5-minute enzyme-inactivation at 80°C in an LA-500 turbidimeter (Eiken Chemical). Results were electronically recorded from the amplification curves in the control unit. An increase in turbidity exceeding 0.1 Optical Density (OD) units per second was scored as positive [22]. In case a sample showed invalid result (questionable curve) or the controls did not show expected results the whole strip of eight tubes were re-analysed.

### Limit of detection

Parasite densities of *P. falciparum* (3D7) laboratory strain cultures were assessed by microscopy and diluted to concentrations of 2000-0.2 p/µL and seeded on filter papers. Detection limits for the Loopamp MALARIA Pan/Pf kit were determined after DNA extractions with Chelex and found to be ≤2 parasites/µL for both Pan and Pf-LAMP.

### Lot testing

Incoming quality check (IQC) for lot release was performed for the two lots of Loopamp MALARIA Pan/Pf detection kit used during the trial with two *P. falciparum* samples of 5 and 50 p/µL, one *P. malariae* sample and three negative samples.

### Re-testing of blood samples with discordant LAMP and PCR results

For the 865 fever patients, DNA samples with discordant Cyt b nested PCR versus Pan and/or Pf-LAMP results were subjected to DNA re-extraction with the Chelex method and re-testing with Cyt b nested PCR and Pan and Pf-LAMP. Any samples which continued to give discordant results were then re-amplified by Cyt b real-time PCR in triplicate. DNA samples from asymptomatic individuals with discordant results between nested PCR versus Pan and/or Pf-LAMP were also re-amplified by Cyt b real-time PCR in triplicate.

### Reference PCR methods

The method used for comparison of PCR versus Pan and Pf-LAMP was Cyt b nested PCR, herein referred to as reference standard. Cyt b real-time PCR corrected nested PCR result was defined as gold standard for evaluation of LAMP in this study.

### Blinding of samples

The person performing the LAMP assays and re-testing of discordant PCR and LAMP samples was blinded to all clinical data and previously obtained results by all other diagnostic methods (microscopy, RDT and PCRs). All samples were anonymized before testing and re-testing.

### Ethical considerations

Sample collections were conducted in accordance with the Declaration of Helsinki and Good Clinical Practice [23], [24] and approved by the Zanzibar Medical Research Ethics Committee (ZAMREC/ST/0021/09) (ZAMREC/0001/JUNE/011) and the Regional Ethics Committee, Stockholm (2009/387-31). All participants in both studies gave written informed consent for their participation. For children, proxy-consents from parents/legal guardians were obtained. The RDT study on fever patients [14] is registered on ClinicalTrials.gov with study identifier “NCT01002066”.

### Data management, sample size calculation and statistical analysis

Data were entered and statistical analyses performed using STATA v.12 (Stata Corp, Texas, USA). The primary endpoint was to assess the accuracy with which the Loopamp MALARIA Pan/Pf detection kit detected *Plasmodium* DNA in fever patients and asymptomatic individuals using dried blood spots collected on filter paper in a malaria pre-elimination setting. The power calculation was based on the assumption that LAMP (both Pan and Pf reactions) should have an average sensitivity of 90% and specificity of 95% when compared with PCR. The proposed sample size, including 121 RDT positive fever patients and 61 asymptomatic PCR positives, would then provide confidence intervals (CIs) within 10% of the sensitivity point estimate (83.6–94.1% and 80.0–95.3%, respectively). Specificity was estimated much more narrowly given that the 748 fever patient RDT negatives and 400 asymptomatic PCR negatives would provide CIs of 93.2–96.3% and 92.4–96.7%, respectively. Sensitivity, specificity, negative (NPV) and positive (PPV) predictive values of the respective Pan and Pf-LAMP assay with corresponding 95% CIs were calculated using real-time PCR corrected nested PCR as gold standard. When calculating the Pf-LAMP sensitivity, specificity, NPV and PPV for the asymptomatic individuals, *P. malariae* mono-infections were not included. The corresponding calculations for Pan-LAMP were based on *P. falciparum* and *P. malariae* mono-infections only, i.e. the eight mixed infections were not included in the calculations. Data were also analysed after stratification by covariates that may influence the diagnostic accuracy of LAMP (e.g. parasite density and presence of non-falciparum species).The immediate commands of cii was used to calculate the 95% CI (STATA v.12). Pairwise determination of non-equivalence between final outcome (including re-extraction) of Pan and Pf-LAMP as well as for Pan-LAMP and Pf-LAMP individually versus nested PCR for detection of parasite DNA was determined by the McNemar test. Statistical significance was defined as p<0.05. Kappa statistics for agreement between the methods were also performed.

## Results

**Figure 1:**
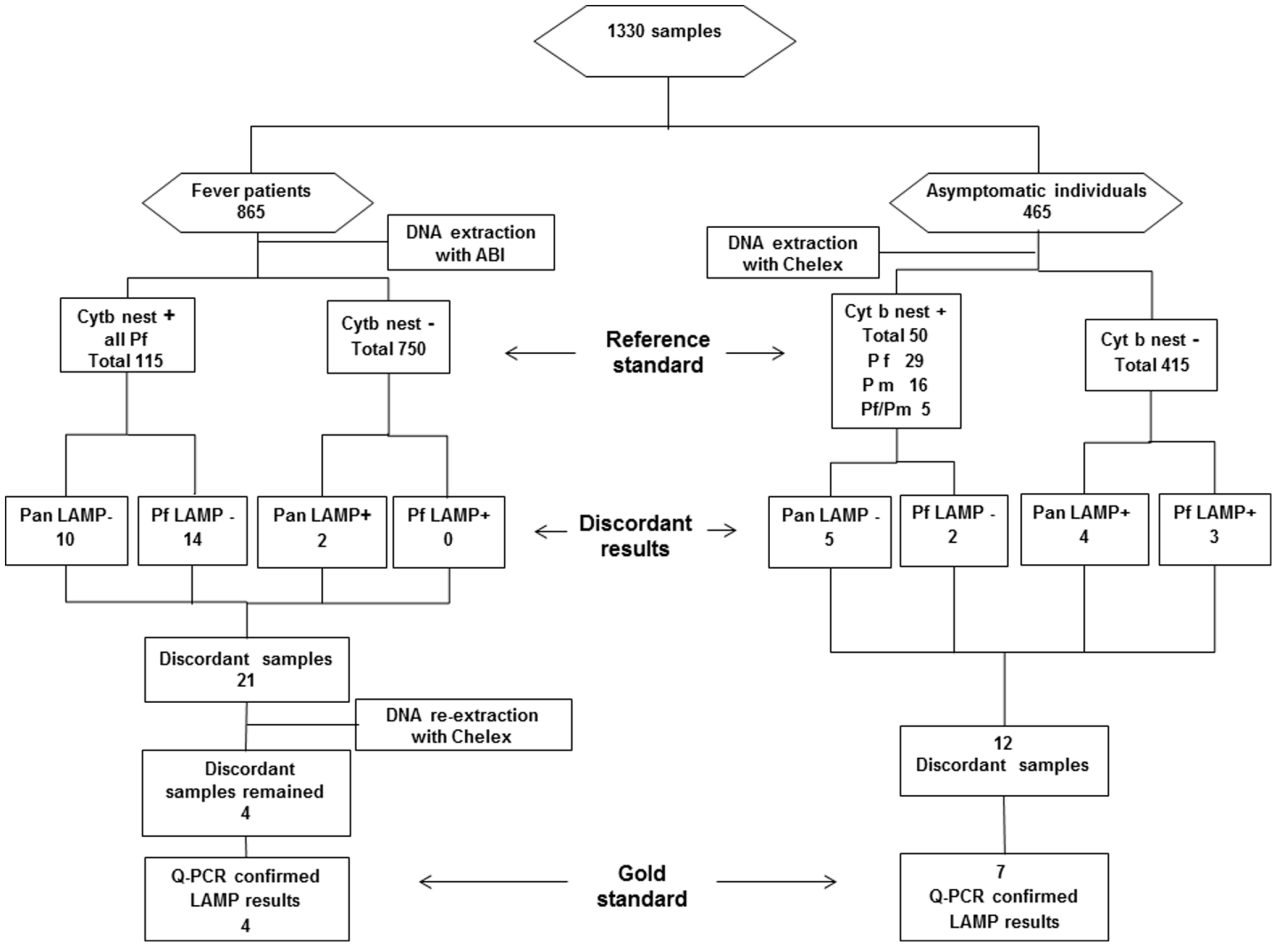
Flow chart of study. Reference standard = Cytochrome B nested PCR. **Gold standard** = Cytochrome B real-time PCR corrected nested PCR.

Baseline characteristics of the included subjects are presented in Table 1 and the study flow is outlined in Figure 1.

**Table 1:** Baseline characteristics of fever patients and asymptomatic individuals.

	Fever patients (n = 865)	Asymptomatic individuals (n = 465)
Median age	19 (2 months-92 years)	14 (1month-85 years)
Sex male %/female %	42/58	40/60
RDT positive *(P. falciparum)*	121	13
RDT positive (non *P. falciparum*)	ND	0
Microscopy positive (all species)	116	ND
Geometric mean parasite density* p/µL	7491 (6–782,400)	ND
PCR positive (all species)	122	65
Geometric mean parasite density** p/µL	ND	10 (0–4972)

*determined by microscopy,

**determined by quantitative PCR,

ND = not done, p/µL = parasites/microliter, values in ( ) = range.

### Fever patients

Overall 115/865 (13.3%) fever patients were positive by Cyt b nested PCR. The geometric mean parasite density (p/µL) determined by blood smear microscopy was 7491 (range 6–782400). All 115 nested PCR positives were *P. falciparum* mono-infections, of which 105 (91%) were detected by Pan-LAMP and 101 (88%) by Pf-LAMP. Two nested PCR negative samples were positive by Pan-LAMP (Figure 1).

There were thus 21 samples with discordant nested PCR versus Pan and/or Pf-LAMP results. Among these, five had negative blood smears and the remaining 16 microscopy positive samples had parasite densities assessed by microscopy ranging from 9 to 5331 p/µL (Table 2). All 21 samples were positive when analysed with real-time PCR in triplicate.

**Table 2:** Fever patient samples with discordant results.

			ABI extracted DNA				Chelex re-extracted DNA			
ID	Parasites/µL*	RDT	Pan LAMP	Pf LAMP	Nested PCR	Real-time PCR	Pan LAMP	Pf LAMP	Nested PCR	Real-time PCR
1	360	+	−	+	+	+	+	+	+	
2	0	+	−	−	+	+	+	+	−	+
3	0	−	−	−	+	+	−	−	−	
4	0	−	+	−	+	+	−	−	−	
5	2691	+	+	−	+	+	+	+	+	
6	0	+	+	−	+	+	+	+	+	
7	1307	+	−	+	+	+	+	+	+	
8	2760	+	+	−	+	+	+	+	+	
9	0	+	+	−	−	+	+	+	−	+
10	3513	+	−	+	+	+	+	+	+	
11	3930	+	+	−	−	+	+	+	−	+
12	340	+	−	−	+	+	+	+	+	
13	9	+	+	−	+	+	+	+	+	
14	319	+	+	−	+	+	+	+	+	
15	233	+	−	−	+	+	+	+	+	
16	0	+	−	−	+	+	+	+	+	
17	1637	+	+	−	+	+	+	+	+	
18	5331	+	−	+	+	+	+	+	+	
19	679	+	−	+	+	+	+	+	−	+
20	2820	+	+	−	+	+	+	+	+	
21	679	+	+	−	+	+	+	+	+	

*Determined by blood smear microscopy, RDT = rapid diagnostic test + positive, − negative.

Outcome summery of fever patient samples with discordant results between Pan and/or Pf-LAMP and nested PCR using ABI extracted DNA and after DNA re-extraction with Chelex.

After repeat PCR and LAMP analysis with Chelex re-extracted DNA from the 21 discordant and 21 randomly selected concordant negative samples as controls, four discordant samples remained, i.e. Pan and Pf-LAMP positive/nested PCR negative. Among these, two samples had parasite densities of 3930 and 679 p/µL, respectively, as assessed by microscopy whereas the other two were negative. All these four samples were again determined positive by Cyt b real-time PCR, see table 2 for detailed results. Two patients negative by Pan and Pf-LAMP were after Chelex-extraction also negative by Cyt b nested PCR. Both samples were negative by microscopy (Table 2). Nested PCR detected 15/21 (71%) of the real-time PCR positive re-extracted samples. All 21 previously concordant negative samples remained negative when re-analyzed.

### Asymptomatic individuals

Overall 50/465 (11%) asymptomatic individuals were positive by Cyt b nested PCR, 29 samples were determined positive for *P. falciparum*, 16 for *P. malariae* and five for *P. falciparum/P. malariae* mixed infections. The geometric mean parasite density (p/µL) determined by qPCR was 10 (range 0–4972). Pan-LAMP was positive for 49 (10.5%) and Pf-LAMP for 38 (8.2%) subjects. There were 12 samples with discordant nested PCR and Pan and/or Pf-LAMP results. Their detailed results including qPCR determined parasite densities are presented in Table 3.

**Table 3:** Asymptomatic individuals with discordant results.

ID	Parasites/µL*	Pan LAMP	Pf LAMP	Nested PCR	Real-time PCR
1	3	+	+	-	Pf
2	3	+	+	-	Pf
3	10	+	+	Pm	Pf/Pm
4	<1	+	+	-	Pf
5	5	−	−	Pf/Pm	Pf/Pm
6	5	+	+	Pm	Pf/Pm
7	2	−	−	Pm	Pm
8	3	+	+	Pm	Pf/Pm
9	1	+	−	-	Pf
10	2	−	−	Pf	Pf
11	1	−	−	Pm	Pm
12	2	−	−	Pm	Pm

*determined by quantitative PCR, Pf = *Plasmodium falciparum* Pm = *Plasmodium malariae*,

P/µL = parasites/microliter, + positive, − negative.

Outcome summary of asymptomatic individuals with discordant results between.

Pan and/or Pf-LAMP and nested PCR using Chelex extracted DNA.

The 12 discordant samples along with the same number of randomly selected concordant negative samples as controls were re-analysed with Cyt b real-time PCR in triplicate. This resulted in another 4 PCR positives among the discordant samples. Among these 54 (50+4) PCR positive samples, the RFLP assay with FspBI digestion showed 33 *P. falciparum*, 13 *P. malariae* and 8 mixed *P. falciparum/P. malariae* infections. Out of the 41 (33+8) *P. falciparum* real-time PCR positive samples, 38 (93%) and 34 (83%) were positive by Pf-LAMP and nested PCR, respectively. Pan-LAMP was positive in 49/54 (91%) of all PCR positive samples and in 32/33 (97%) and 10/13 (77%) of the PCR determined *P. falciparum and P. malariae* mono-infections, respectively. Nested PCR was positive for all 13 *P. malariae* samples. There was no significant difference in parasite densities between the three Pan-LAMP negative (range 1–2 p/µL) versus the ten Pan-LAMP positive (range 0–3 p/µL) *P. malariae* samples (p≥0.05). Pan and Pf-LAMP both detected seven, whereas nested PCR detected five of the eight samples with mixed *P. falciparum*/*P. malariae* infections. All 12 previously concordant negative samples remained negative when re-analysed.

### Sensitivities and Specificities for LAMP

#### Fever patients

The sensitivity, specificity and predictive values for parasite detection in ABI-extracted samples for Pan and Pf-LAMP versus real-time PCR corrected nested PCR (gold standard) are presented in Table 4. Final outcome for Pan and Pf-LAMP among fever patients including the results from the analysis using Chelex re-extracted DNA samples (823+42) and the respective kappa-values are also presented in Table 4. Statistical equivalence between the performance of Pan versus Pf-LAMP and between Pan-LAMP and Pf-LAMP individually versus nested PCR for detection of *P. falciparum* DNA revealed p-values of 1.00, 0.13 and 0.13, respectively.

**Table 4:** Sensitivities, specificities, positive and negative predictive values and kappa analysis.

Fever patients ABI-extracted DNA (n = 865)					
	Sensitivity % (95% CI)	Specificity % (95% CI)	PPV % (95% CI)	NPV % (95% CI)	Kappa value
Pan-LAMP	91.5 (84.8–95.8)	100 (99.5–100)	100 (96.6–100)	98.7 (97.6–99.4)	0.95
Pf-LAMP	86.3 (78.7–92.0)	100 (99.5–100)	100 (96.4–100)	97.9 (96.6–98.8)	0.92

Fever patients: including Chelex re-extracted DNA (n = 823+42)					
	Sensitivity % (95% CI)	Specificity % (95% CI)	PPV % (95% CI)	NPV % (95% CI)	Kappa value
Pan-LAMP	98.3 (94.0–99.8)	100 (99.5–100)	100 (96.8–100)	99.7 (99.0–100)	0.99
Pf-LAMP	98.3 (94.0–99.8)	100 (99.5–100)	100 (96.8–100)	99.7 (99.0–100)	0.99

Asymptomatic individuals Chelex extracted DNA (n = 465)					
	Sensitivity % (95% CI)	Specificity % (95% CI)	PPV % (95% CI)	NPV % (95% CI)	Kappa value
Pan-LAMP all species	90.7 (79.7–96.9)	100 (99.1–100)	100 (92.7–100)	98.8 (97.2–99.6)	0.95
Pan-LAMP *P.f*.*	97 (84.2–99.9)	100 (99.1–100)	100 (89.1–100)	99.8 (98.7–100)	0.97
Pan-LAMP P.m.**	76.9 (46.2–95)	100 (99.1–100)	100 (69.2–100)	99.3 (97.9–99.9)	0.89
Pf-LAMP P.f.***	92.7 (80.1–98.5)	100 (99.1–100)	100 (90.7–100)	99.3 (98.0–99.9)	0.96

P.f.* = *P. falciparum* mono infections (n = 33), P.m.** = *P.malariae* mono infections (n = 13), P.f.*** *P. falciparum* mono and mixed.

infections (n = 41), CI = confidence interval, PPV = positive predictive value, NPV = negative predictive value.

Sensitivities, specificities, positive and negative predictive values and kappa analysis for detection of malaria DNA from fever patients and asymptomatic individuals with Pan and Pf-LAMP versus gold standard (real- time PCR corrected Cytochrome b nested PCR).

#### Asymptomatic individuals

The sensitivity, specificity and predictive values for parasite DNA detection for the respective Pan and Pf-LAMP compared with the defined PCR gold standard are presented in Table 4. Statistical equivalence between the performances of Pan versus Pf-LAMP and between Pf-LAMP versus nested PCR for detection of *P. falciparum* among asymptomatic individuals revealed no significant difference, with p-values of 1.00 and 0.29, respectively. Similarly, the performance of Pan-LAMP versus nested PCR for detection of all malaria positives also showed equivalence (p = 1.00).

## Discussion

The Loopamp MALARIA Pan/Pf detection kit evaluated in this study revealed high diagnostic accuracy both with Pan and Pf-LAMP for parasite DNA detection among fever patients and asymptomatic individuals from filter paper blood samples collected in Zanzibar. This is, to our knowledge, the first evidence of high diagnostic accuracy of LAMP using parasite DNA extracted from minute blood volumes spotted on filter paper from fever patients and importantly asymptomatic low-density parasitaemias. These data support LAMPs potential role for improved passive and active malaria case detection in pre-elimination settings.

The LAMP method has previously been evaluated against microscopy and PCR among symptomatic patients suspected of having malaria infection, already diagnosed malaria cases and from samples obtained from malaria cultures using larger blood volumes [10], [11], [12], [22], [25] showing sensitivities and specificities >90%. Importantly, the present study shows similar high diagnostic accuracy from asymptomatic individuals using minute blood volumes preserved on filter paper. This is in agreement with a recently published study using a RealAmp assay with high diagnostic accuracy with DNA extracted from dried blood spots from asymptomatic individuals in Thailand, although on a very small number of positive samples [26].

The Loopamp MALARIA Pan/Pf detection kit has previously been shown to be stable, user-friendly and robust [22]. The kit is also considered safe with minimal risk of contamination [12], [27]. The high amplification capacity provides highly sensitive parasite detection with either a turbidimeter or under UV-light after 40 minutes incubation. Moreover, the high amplification capacity of LAMP makes the obtained results easy to interpret; with a few parasites per microliter of blood generating equally high turbidity or fluorescence as high-density parasitaemias [12].

In the present study, LAMP performed less well on DNA samples extracted with an automated ABI platform. However, LAMP analysis of the same blood samples, but DNA extracted with Chelex resulted in an excellent agreement between PCR and LAMP and importantly, we did not find any false positive LAMP results.

Among asymptomatic individuals, both Pan and Pf-LAMP had high sensitivities in identifying infections with low parasite densities. LAMP appeared also to be a better tool to identify mixed *P. falciparum*/*P. malariae* infections compared to nested PCR. Moreover, Pan-LAMP appeared to have slightly lower sensitivity in detecting low-density *P. malariae* infections compared to nested PCR, on the other hand LAMP was superior for *P. falciparum* detection. Our results are in accordance with a previous evaluation of Pan and Pf-LAMP for detection of the various *Plasmodium* species showing a detection limit of 2–5 p/µL for all human malaria species [12]. Similarly with the results retrieved from fever patient samples, no false positive LAMP result (specificity and PPV 100%) was detected among the asymptomatic individuals.

Our results show that the Loopamp MALARIA Pan/Pf detection kit may represent a promising opportunity for improved malaria case detection in screen and treat activities within malaria pre-elimination settings. Considering that the relative importance of *non-falciparum* infections appear to increase in such areas [2], [7] screening with Pan-LAMP only, which also has a slightly lower detection limit compared to Pf-LAMP (5 versus 7.5 DNA copies/test) [21], may represent a cost effective strategy followed by Pf-LAMP analysis of Pan-LAMP positive samples. However, there is a need for vivax-specific LAMP assays especially in areas outside Africa where *P.vivax* infections predominate [26]. Previous studies have demonstrated high diagnostic accuracy of LAMP with fresh or frozen blood [10], [11], [12], [22], [25]. The present study provides evidence that the LAMP kit performs equally well with blood samples from finger pricks collected and stored on filter paper, a low-cost and practical solution for population screening purposes. However, simpler and faster sample preparation for LAMP has been evaluated in the field [5]. Such simplification of sample processing and thus at low cost and improved throughput of high number of samples represents a priority if LAMP is going to be a useful tool in areas aiming at malaria elimination. Importantly, and based on our data, future field evaluations of the LAMP assays for detection of asymptomatic low-density parasite carriers need to be conducted. Further studies on the impact on overall transmission of low-density parasite detection by LAMP followed by adequate treatment need to be conducted.

### Limitations

In this study two sets of DNA samples previously extracted with two different methods and stored at −20°C were used. Samples from fever patients were extracted with a column-based ABI method, a high throughput set-up used in our laboratory at the time of the trial [14], [17]. This method has been replaced by the Chelex-100 extraction method in our laboratory. The discordant samples between nested PCR and LAMP were therefore re-extracted using the latter method. The concordant ABI extracted PCR positive and negative samples which were not re-extracted, were earlier investigated with several nested and real-time PCR methods [14].

Long storage of frozen DNA extracted with the ABI method, in combination with several freeze-thawing episodes may have influenced the DNA quality suitable for the LAMP reaction more than for ordinary PCRs since several samples with microscopically detectable parasitaemias were negative for Pan and/or Pf-LAMP, but positive for Cyt b nested and real-time PCR. When recently extracted DNA with the Chelex method was used both Pan and Pf-LAMP showed even higher sensitivity than nested PCR.

### Conclusion

Both components, Pan- and Pf-detection, of the Loopamp MALARIA kit evaluated in this study revealed high diagnostic accuracy for parasite detection among both fever patients and asymptomatic individuals. This study provides, to our knowledge, the first published evidence of high diagnostic accuracy of LAMP for parasite detection from minute blood volumes spotted on filter paper from asymptomatic individuals in a population where elimination strategies such as focal screening and treatment may be of value, particularly when blood sampling on filter paper from capillary finger pricks would be advantageous for practical and logistical reasons. These data support LAMPs potential role for the implementation of active case detection activities in malaria pre-elimination settings.
